# First experience of nDASEK with heads-up Surgery: A case report: Erratum

**DOI:** 10.1097/MD.0000000000012287

**Published:** 2018-08-21

In the article, “First experience of nDASEK with heads-up Surgery: A case report”,^[1]^ which appeared in Volume 96, Issue 19 of *Medicine*, nDSAEK was spelled incorrectly in the title as nDASEK and should be “First experience of nDSAEK with heads-up surgery: A case report.”
